# A Sensitive DNA Enzyme-Based Fluorescent Assay for Bacterial Detection

**DOI:** 10.3390/biom3030563

**Published:** 2013-08-20

**Authors:** Sergio D. Aguirre, M. Monsur Ali, Bruno J. Salena, Yingfu Li

**Affiliations:** 1 Department of Biochemistry and Biomedical Sciences, McMaster University, 1280 Main St. W., Hamilton, ON L8S 4K1, Canada; E-Mails: sergio.aguirre454@gmail.com (S.D.A); mmonsurali@gmail.com (M.M.A.); 2 Dvision of Gastroenterology, Department of Medicine, McMaster University, 1280 Main St. W., Hamilton, ON L8S 4K1, Canada; E-Mail: salenab@mcmaster.ca; 3 Department of Chemistry and Chemical Biology, McMaster University, 1280 Main St. W., Hamilton, ON L8S 4K1, Canada; 4 Michael G. DeGroote Institute for Infectious Disease Research, McMaster University, 1280 Main St. W., Hamilton, ON L8S 4K1, Canada

**Keywords:** bacterial detection, DNAzyme, fluorescence, biosensor, *Escherichia coli*

## Abstract

Bacterial detection plays an important role in protecting public health and safety, and thus, substantial research efforts have been directed at developing bacterial sensing methods that are sensitive, specific, inexpensive, and easy to use. We have recently reported a novel “mix-and-read” assay where a fluorogenic DNAzyme probe was used to detect model bacterium *E. coli*. In this work, we carried out a series of optimization experiments in order to improve the performance of this assay. The optimized assay can achieve a detection limit of 1000 colony-forming units (CFU) without a culturing step and is able to detect 1 CFU following as short as 4 h of bacterial culturing in a growth medium. Overall, our effort has led to the development of a highly sensitive and easy-to-use fluorescent bacterial detection assay that employs a catalytic DNA.

## 1. Introduction

Infectious agents, such as foodborne pathogens, have caused numerous large-scale and costly outbreaks in the human history and will continue to be a major public health threat and financial burden for our society [1,2,3,4]. Early detection of pathogens, as the first step to prevent such outbreaks, has become increasingly more important today because the globalization of commerce and speedy travel have significantly increased the rate and breadth of the spread of infectious agents. Thus, the demand for faster, simpler, less expensive and more reliable pathogen testing methods has become ever greater. 

Although the traditional culture method continues to be the ‘gold standard’ for bacterial detection, it is time-consuming and requires days or even weeks to complete (depending on the specific pathogen in question) [5]. Modern methods take advantage of well-established biomolecular techniques, such as polymerase chain reaction (PCR) and immunoassay (where an antibody is used as molecular recognition element), to achieve faster and more sensitive pathogen detection [5,6,7,8,9,10,11]. Despite the popularity of these techniques, they also come with certain drawbacks, such as the need for costly instrumentation and highly trained personnel to isolate or purify relevant targets (DNA for PCR and proteins for immunoassays). Thus, the entire test using such methods often still needs one or more days to complete. Detection sensitivity (for immunoassay) and tendency to generate false-positive results (for PCR) are also issues of concerns. For these considerations, we recently began to examine the utility of RNA-cleaving fluorogenic DNAzyme (RFD) probes for bacterial detection [12,13,14]. RFDs can be isolated from random-sequence DNA pools to perform three linked functions: ligand binding, catalysis and fluorescence generation. Each RFD cleaves a synthetic nucleic acid substrate containing a single ribonucleotide as the cleavage site embedded in a DNA sequence, and the cleavage site is located between two nucleotides modified with a matching pair of fluorophore and quencher [12,13,14,15,16,17,18,19,20,21]. Because of these two features, these reporter molecules emit an increasing level of fluorescence when they carry out the catalytic cleavage of the RNA linkage. In other words, the cleavage event results in separation of the fluorophore from the quencher, accompanied by the increase of fluorescence intensity in real time. 

More recently, we developed a method of isolating novel DNAzyme probes against the crude extracellular mixture (CEM) left behind by a specific type of bacteria in their environment or in the media they are cultured [12]. The CEM is rich in diverse targets, including small molecules and proteins. Thus the use of the crude mixture as the complex target to conduct *in vitro* selection [22,23,24] experiment circumvents the tedious process of purifying and identifying a suitable target from the microbe of interest for biosensor development, and provides a subsequent assaying procedure that is simple because it does not require steps to purify a target of interest. Using this approach, we have isolated an RFD that cleaves its substrate only in the presence of the CEM produced by *E. coli* (CEM-EC) [12]. This *E. coli*-sensing RFD, named RFD-EC1, was found to be highly selective to CEM-EC but nonresponsive to CEMs from many other Gram-negative and Gram-positive bacteria. We have also shown that the DNAzyme-based assay is capable of reporting the presence of a single *E. coli* cell after 12 h of culturing. These experiments have illustrated the utility of RFDs as fluorogenic bacterial indicators. In this work we carried out a thorough investigation to characterize this bacterial detection system with a goal to further improve the detection sensitivity.

## 2. Results and Discussion

### 2.1. Establishing a *Trans*-Acting DNAzyme

**Figure 1:**
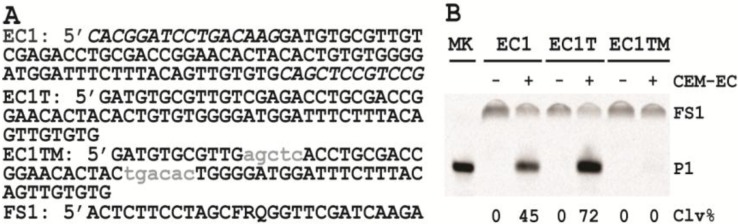
Design of *trans*-acting DNAzymes. (**A**) The sequences of EC1, EC1T, EC1TM and FS1. EC1 is the full length DNAzyme including two primer binding sites (nucleotides in italic) for polymerase chain reaction used in the original *in vitro* selection experiment. EC1T is the shortened version of EC1 with deleted primer binding sites. EC1TM is a mutant of EC1T wherein the nucleotides shown as lower-case letters are altered. The substrate FS1 contains an adenosine ribonucleotide (R) flanked by a fluorescein-dT (F) and a DABCYL-dT (Q). (**B**) dPAGE analysis of the cleavage reaction mixtures of FS1 with EC1, EC1T, or EC1TM in the absence (−) and presence (+) of CEM-EC. P1 represents the 5’-cleavage product, which can be observed by fluorescence scan as it contains the F unit. MK (marker) is a sample of FS1 fully cleaved by NaOH. Clv% for each sample was calculated following our previously reported method [20].

Our previously reported RFD-EC1 is a *cis*-acting DNAzyme that cleaves a covalently attached substrate. However, a *trans*-acting DNAzyme where the DNAzyme cleaves a detached substrate has an additional advantage such as ease-of-synthesis, thus lowering the cost and labor. Synthesis of long DNA chain modified with fluorophore, quencher and ribonucleotide is associated with lower yields and higher costs. Therefore, in this study, we first examined the possibility of converting it into a *trans*-acting catalyst by detaching the substrate portion of the sequence, FS1, from the DNAzyme portion, EC1 (Figure 1A). We found that EC1 was indeed able to cleave FS1 in *trans*, even at 1:1 ratio (50 nM each of EC1 and FS1), in a CEM-EC dependent manner (Figure 1B). Note that the reaction mixtures were analyzed by denaturing polyacrylamide gel electrophoresis (dPAGE). 

We next tested a second *trans* construct, named EC1T (Figure 1A), by truncating 28 nucleotides from the two ends of EC1 (italic letters, Figure 1A) that were used as the primer-binding sites for polymerase chain reaction during the original *in vitro* selection experiment. Interestingly, EC1T was found to be considerably more active than EC1 (comparing Lanes 3 and 5, Figure 1B; *i.e.*, 45% *vs*. 72%). As a control, we also tested a mutant sequence, EC1TM, with 10 nucleotides (lower-case letters, Figure 1A) mutated from EC1T. These mutations rendered EC1TM completely inactive in the presence of CEM-EC (Figure 1B).

### 2.2. Comparing DNAzyme Activity Using Crude Extracellular Mixture (CEM) and Crude Intracellular Mixture (CIM) of *E. coli*

**Figure 2:**
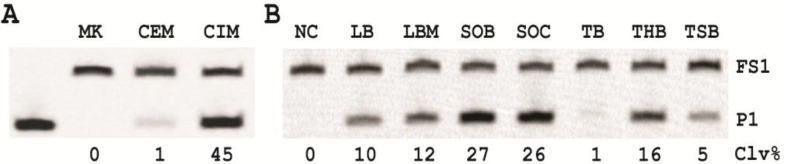
Cleavage reactions of EC1T/FS1 with (**A**) crude extracellular mixture (CEM)-EC and CIM-EC and (**B**) crude intracellular mixture (CIM)-EC collected from *E. coli* cells grown in various culture broths. NC is a negative control where the reaction was conducted in the absence of CEM-EC and CIM-EC. Each reaction mixture was analyzed by 10% dPAGE, followed by fluorimaging. NC: negative control where the reaction was conducted in RB without CEM-EC or CIM-EC.

The original DNAzyme RFD-EC1 was isolated to cleave in the presence of CEM of *E. coli*. We hypothesized that the target that activates the DNAzyme might be more abundant inside the cellular environment. To test this idea, we made an *E. coli* culture and used it to prepare the CEM-EC and CIM-EC as follows: the cells were precipitated by centrifugation and the supernatant was taken as the CEM-EC. The cell pellet was re-suspended in the reaction buffer, heat-treated, and then centrifuged; the remaining supernatant was taken as the CIM-EC (see experimental section for details). The CEM-EC and CIM-EC were then used to induce the cleavage activity of EC1T towards FS1, and the results are illustrated in Figure 2A. It is clear that the CIM-EC indeed contained a much higher amount of the target than the CEM-EC as it induced much stronger cleavage of FS1 by EC1T (45% *vs.* 1%). Note that much lower cleavage in this experiment with CEM-EC is due to the shorter culture time (7 h) with low number of *E. coli* cells (50,000 colony forming units). For the remaining experiments, the CIM-EC was used as the target of interest.

### 2.3. Searching for an Optimal Culture Broth

We next investigated the effect of bacterial growth media on the quality of CIM (as measured by the cleavage activity of EC1T/FS1) in order to establish an optimal culture broth. Seven common growth media were chosen for this analysis and they were: Luria Bertani (LB), Terrific Broth (TB), Todd-Hewitt (TH), Lysogeny Broth Miller (LBM), Tryptic Soy Broth (TSB), Super Optimal Broth (SOB) and Super Optimal Broth with Catabolic repressor (SOC). 250 *E. coli* cells were allowed to grow in 1 mL of each broth for 7 h at 37 °C, from which CIM was prepared and used to induce the cleavage of EC1T/FS1; the results are illustrated as Figure 2B. The CIMs from SOB and SOC produced the highest activity (~26% cleavage), followed by those from LB, LBM, and THB (10–16%). The CEMs from TSB and TB were least effective (≤5%). Based on these results, SOB was chosen as the broth for the remaining experiments.

### 2.4. Effects of Divalent Metal Ions

**Figure 3:**
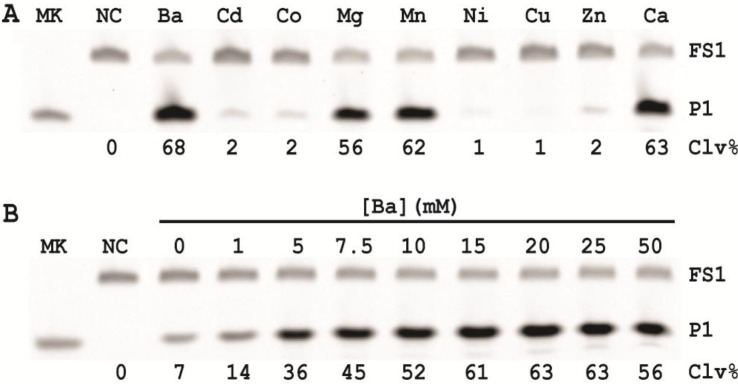
(**A**) Cleavage activity of EC1T/FS1 in the presence of CEM-EC and various divalent metal ions. (**B**) Effect of the Ba^2+^ concentration.

Divalent metal ions play crucial roles in catalytic functions of DNAzymes and it has been shown that different metal ions can significantly affect the catalytic activity of a DNAzyme [25,26,27,28]. For example, 8-17, a well-studied RNA-cleaving DNAzyme, exhibits the highest activity in presence of lead ions even though it was originally isolated using Mg^2+^ [29] or Zn^2+^ [30]. A recent study has revealed that Pb^2+^ promotes the most favorable folding of 8-17 [31]. Therefore, we sought to compare the effects of various divalent metal ions on the activity of our *E. coli*-sensing DNAzyme although the original DNAzyme RFD-EC1 was obtained by *in vitro* selection in the presence of 15 mM MgCl_2_ [12]. Nine different divalent metal ions were tested and they were: Ba^2+^, Cd^2+^, Co^2+^, Mg^2+^, Mn^2+^, Ni^2+^, Cu^2+^, Zn^2+^, and Ca^2+^; the results are given in Figure 3A. We found that Ba^2+^, Ca^2+^, Mg^2+^ and Mn^2+^ all induced a robust cleavage activity of the DNAzyme (causing 56–68% of cleavage). In contrast, Cd^2+^, Co^2+^, Ni^2+^, Cu^2+^, and Zn^2+^ resulted in weak cleavage (1–2%). It is possible that Ba^2+^ Mg^2+^, Mn^2+^ and Ca^2+^ fit into the catalytic core better than the other divalent metal ions. However, this should be experimentally verified. 

It is noteworthy that we have previously shown that Mn^2+^ exhibits potent fluorescence quenching effect, resulting in significantly reduced signal magnitude when the fluorescence intensity is measured in a fluorimeter [32]. We also found a similar effect of Mn^2+^ in our assay (data not shown). In contrast, Ba^2+^ produced no quenching effect. This observation indicates that Ba^2+^ is a more suitable divalent metal ion for our assay. Thus, Ba^2+^ was chosen for further experiments. In order to establish the optimal Ba^2+^ concentration we investigated the effect of Ba^2+^ concentration on EC1T’s activity. The data presented in Figure 3B indicates that the catalytic activity of EC1T reaches a plateau at 15 mM Ba^2+^. 

### 2.5. Varying Reaction Temperature

**Figure 4:**
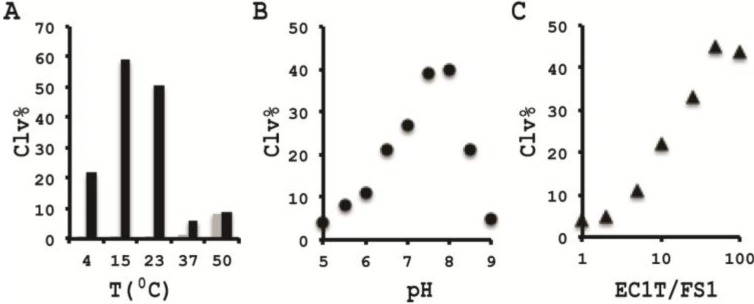
Cleavage activity of EC1T/FS1 with varying temperature (**A**), pH (**B**), and EC1T/FS1 ratio (**C**). The data are the average of two independent experiments.

We examined the cleavage activity of EC1T/FS1 at different temperatures and the results are provided in Figure 4A. A robust cleavage activity was observed at both 15 and 23 °C. In contrast, reduced activity was observed when the reaction temperature was decreased to 4 °C or increased to 37 °C and 50 °C. Interestingly, although CIM was absolutely required to induce the cleavage at 4, 15 and 23 °C, EC1T can cleave FS1 in the absence of CIM at both 37 °C and 50 °C (grey bars in Figure 4A). Since room temperature is the most ideal condition to conduct assays avoiding the requirements of heating and cooling system, we chose 23 °C as the reaction temperature for the remaining experiments.

### 2.6. pH Effect

We next examined the activity of EC1T/FS1 when the reaction pH was varied between 5.0 and 9.0; the results are shown in Figure 4B. Although EC1T was able to cleave FS1 in the entire pH range tested, the highest activity was observed at pH 7.5–8.0. Since the original DNAzyme was isolated at pH 7.5, it is not surprising that EC1T exhibits such a narrow pH preference. 

### 2.7. DNAzyme/Substrate Ratio

We also examined the cleavage activity at different ratios of EC1T/FS1. For this experiment, the concentration of FS1 was kept at 50 nM while the DNAzyme concentration was changed from 0 to 5 µM; the results are shown in Figure 4C. The cleavage activity reached the plateau at a ratio of 50:1. Thus, this ratio was used for the remaining experiments.

### 2.8. Specificity

**Figure 5:**
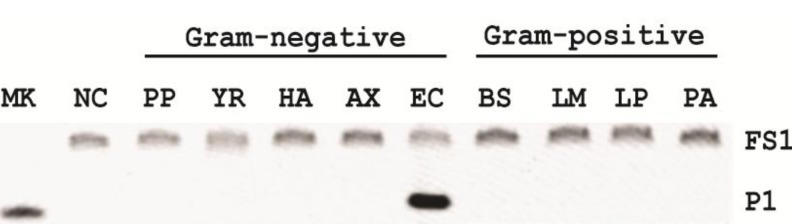
Specificity of EC1T/FS1 for various gram-negative and gram-positive bacteria. PP: *Pseudomonas peli*, YR: *Yersinia rukeri*, HA: *Hafnea alvei*, AX: *Achromobacter xylosoxidans*, EC: *Escherichia coli*, BS: *Bacillus subtilis*, LM: *Leuconostoc mesenteroides*, LP: *Lactobacillus planturum*, PA: *Pediococcus acidilactici*.

With the significant changes of the reaction conditions, we wondered if EC1T was still able to maintain its specificity for *E. coli*. Four other gram-negative bacteria and four gram-positive bacteria were arbitrarily chosen for comparison. Each bacterium was cultured in SOB for a different period of time until the OD_600_ (optical density at 600 nm) of each culture reached ~1. The CIM was then prepared and tested with EC1T/FS1 under the optimal reaction buffer (50 mM HEPES, pH 7.5, 150 mM NaCl and 15 mM BaCl_2_, room temperature, EC1T/FS1 = 50/1). None of the CIMs from other bacteria was able to induce cleavage (Figure 5), indicating that EC1T/FS1 retained the specificity for *E. coli*.

### 2.9. Detection Sensitivity

**Figure 6:**
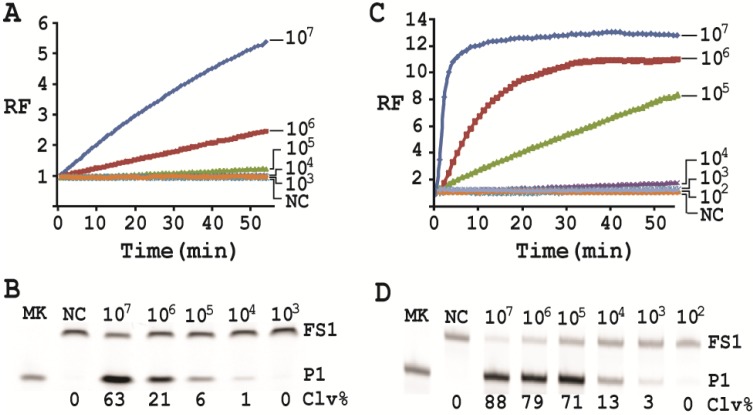
Sensitivity test. (**A**) Real-time fluorescence monitoring and (**B**) dPAGE analysis of EC1T/FS1 in the presence of CIMs prepared from 10^3^–10^7^ *E. coli* cells. (**C**) and (**D**) Similar experiments using RNA-cleaving fluorogenic DNAzyme (RFD-EC1) with CIMs prepared from 10^2^–10^7^ *E. coli* cells. The data in (A) and (C) are the average of two independent experiments.

To test the detection sensitivity of EC1T/FS1, we prepared a series of *E. coli* stock solutions from which CIM samples were prepared as described in experimental section. These samples were then assessed for inducing the cleavage of EC1T/FS1 under the optimal reaction condition established above. These reactions were monitored in a fluorimeter in real time for 60 min (Figure 6A). The reaction mixtures were also analyzed by dPAGE (Figure 6B). We found that the fluorimeter method was able to detect 10^5^ cells while the dPAGE method can detect 10^4^ cells.

We also tested the detection sensitivity of the original *cis*-acting DNAzyme RFD-EC1 using the optimal reaction condition. Interestingly, RFD-EC1 showed better sensitivities: the fluorimeter method can detect 10^4^ cells (Figure 6C) while the dPAGE method was able to detect 10^3^ cells (Figure 6D).

### 2.10. Detection of a Single Cell via Culturing

**Figure 7:**
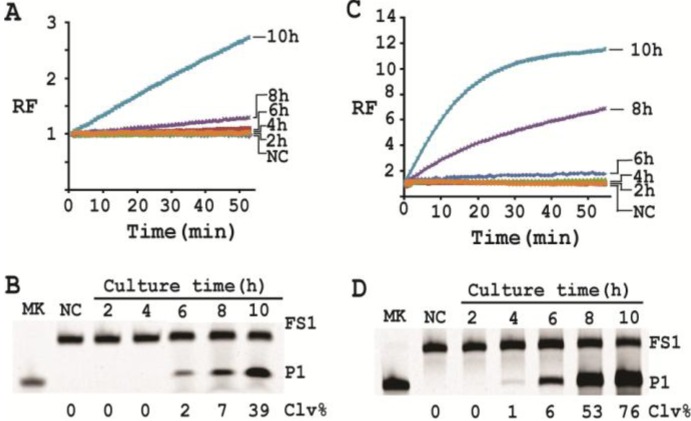
Culturing time required to detect a single *E. coli* cell (1 CFU). (**A**) Monitoring fluorescence of EC1T/FS1 with CIMs prepared from samples taken after a culturing time of 2, 4, 6, 8 and 10 h. (**B**) dPAGE analysis of the reaction mixtures in (A). (**C**) and (**D**) are equivalent experiments in which RFD-EC1 was used to replace EC1T/FS1. The data in (A) and (C) are the average of two independent experiments.

Finally we determined the time required to enrich a single live bacterium (*i.e.*, one colony forming unit or 1 CFU) via culturing in SOB. Following a previous protocol [12], we inoculated a single *E. coli* cell in SOB and cultured for 2, 4, 6, 8 and 10 h at 37 °C. CIMs were prepared for the samples collected at each time point and tested with both *trans* and *cis* constructs. These samples were then assessed for inducing the cleavage of EC1T/FS1 under the optimal reaction condition. Each reaction was examined both in a fluorimeter (Figure 7A) and by dPAGE (Figure 7B). Using EC1T/FS1, 8 h of culturing was sufficient for detection by the fluorimeter method (Figure 7A) and 6 h by dPAGE method (Figure 7B). Using RFD-EC1, however, only 6 h and 4 h of culturing were required to achieve the detection by the fluorimeter (Figure 7C) and dPAGE (Figure 7D) method, respectively. The lower activity of EC1T/FS1 in comparison to the *cis*-acting RFD-EC1 might be due to the weakened interaction between enzyme and substrate strands when they were separated from each other. 

## 3. Experimental Section

### 3.1. Synthesis and Purification of Oligonucleotides

The standard DNA oligonucleotides (EC1, EC1T, EC1TM and EC1LT) were purchased from Integrated DNA Technologies (IDT, Coralville, IA, USA) and purified by 10% denaturing polyacrylamide gel electrophoresis (dPAGE). The modified oligonucleotide FS1 was acquired from W. M. Keck Oligonucleotide Synthesis Facilities (Yale University, New Haven, CT, USA), deprotected and purified by 10% dPAGE following a previously reported protocol [15]. 

### 3.2. Enzymes and Chemical Reagents

T4 DNA ligase and T4 polynucleotide kinase (PNK) were purchased from MBI Fermentas (Burlington, ON, Canada). Tryptone and yeast extract was acquired from BD Biosciences (Mississauga, ON, Canada). All other chemical reagents were purchased from Sigma-Aldrich (Oakville, ON, Canada) and were used without further purification. 

### 3.3. Growth Media

Luria Bertani (LB), Terrific Broth (TB), and Todd-Hewitt (TH) were purchased from Sigma-Aldrich. Lysogeny Broth Miller (LBM) was obtained from EMD Canada (Mississauga, ON, Canada). Tryptic Soy Broth (TSB) was acquired from BD Biosciences. Super Optimal Broth (SOB) and Super Optimal Broth with Catabolic repressor (SOC) were made in house. SOB contains 2% (w/v) tryptone, 0.5% (w/v) yeast extract, 10 mM NaCl and 2.5 mM KCl. SOC has the same ingredients as SOB but also contains 20 mM glucose and 10 mM MgCl_2_. 

### 3.4. Preparation of *Cis*-Acting RFD-EC1

RFD-EC1 was generated by template-mediated ligation of FS1 to EC1. In brief, 200 pmol of FS1 were treated with 1× PNK buffer A (MBI Fermentas), 1 mM ATP and 20 U (units) of PNK for 30 min at 37 °C (reaction volume = 50 μL). The reaction was quenched by heating at 90 °C for 5 min. Equimolar EC1 and EC1LT (5’-CTAGG AAGAG TCGGA CGGAG CTG; the ligation template) were then added to this solution and was heated at 90 °C for 30 s and cooled to room temperature for 10 min. Afterwards, 10 μL of 10× T4 DNA ligase buffer (MBI Fermentas), 39 μL of deionized distilled water (ddH_2_O) and 1 μL of T4 DNA ligase (10 U/μL) were added. After incubation at room temperature (RT) for 2 h, the ligated EC1-FS1 was purified by 10% dPAGE.

### 3.5. Bacterial Cells

Gram-negative bacteria *Pseudomonas peli*, *Yersinia rukeri*, *Hafnea alvei*, and *Achromobacter xylosoxidans* were donated by Dr. Gerard Wright (Micheal G. DeGroote Institute for Infectious Disease Research, McMaster University). Gram-positive bacteria *Leuconostoc mesenteroides*, *Lactobacillus planturum* and *Pediococcus acidilactici* (PA) were gifts from Dr. Brian Coombes and Dr. Russel Bishop (Department of Biochemistry and Biomedical Sciences, McMaster University). *E. coli* K12 (MG1655) and *Bacillus subtilis* 168 are regularly maintained in our laboratory.

### 3.6. Comparison of the Cleavage Activity of EC1, EC1T and EC1TM in the Presence of CEM-EC

*E. coli* was plated onto a TSB agar (1.5%) plate and grown for 14 h at 37 °C. A single colony was taken and inoculated into 2 mL of TSB and grown for 14 h at 37 °C with shaking at 250 rpm. A 1% fresh culture was made by re-inoculating 20 µL of the above culture into 2 mL of TSB. The re-inoculation was allowed to grow at 37 °C with shaking at 250 rpm until the culture reached an OD_600_ of ~1. 1 mL of this culture was centrifuged at 11,000 g for 5 min at room temperature; the supernatant was taken as the crude extracellular mixture (CEM-EC) and stored at −20 °C.

For each candidate DNAzyme construct, two reactions were set up, a control and a test. For the test, 25 μL of 2× reaction buffer (2× RB; 100 mM HEPES, 300 mM NaCl, 30 mM MgCl_2_, pH 7.5) was mixed with 23 μL of the CEM-EC prepared above, 1 μL of 2.5 μM FS1 and 1 μL of 2.5 μM EC1, EC1T or EC1TM. For the control, TSB was used to substitute the CEM-EC. Each reaction mixture was incubated at RT for 60 min, followed by quenching with 5 μL of 3 M NaOAc (pH 5.5) and 135 μL of cold ethanol. DNA was recovered by centrifugation and analyzed by 10% dPAGE. DNA bands in the gel were visualized by Typhoon 9200 (GE Healthcare) and quantified by ImageQuant software (Molecular Dynamics). 

### 3.7. Comparison of the Cleavage Activity of EC1T in the Presence of CEM-EC and CIM-EC

100 μL of 50,000 CFU/mL glycerol stock of *E. coli* was inoculated into 2 mL of TSB and grown at 37 °C for 7 h with shaking at 250 rpm. 1 mL of this culture was centrifuged at 11,000 g for 5 min at room temperature; the supernatant was taken as the CEM-EC for this experiment. The cell pellet was suspended in 200 µL of 1× RB and heated at 50 °C for 15 min. The heat-treated cell suspension was then centrifuged at 11,000 g for 5 min at RT. The clear supernatant was taken as the CIM-EC for the experiment. 

The cleavage reaction with the CEM-EC was carried out by mixing 25 μL of 2× RB, 23 μL of the CEM-EC prepared above, 1 μL of 2.5 μM FS1 and 1 μL of 2.5 μM EC1T. The reaction concerning the CIM-EC was conducted by mixing 41 μL of 1× RB, 5 μL of CIM-EC, 1 μL of 2.5 μM FS1, 1 μL of 2.5 μM EC1T and 2 μL of 2× RB (note that the CIM-EC was made by suspending the cell pellet from originally 1 mL of *E. coli* culture in 200 μL of 1× RB, which translates into a concentrating factor of 5). A control experiment without the CEM-EC and CIM-EC was also conducted. Each reaction mixture was incubated at RT for 60 min, followed by 10% dPAGE analysis as described above. 

### 3.8. Comparison of the Cleavage Activity of EC1T in the Presence of CIM-EC Obtained from *E. coli* Grown in Various Growth Media

100 μL of 2500 CFU/mL glycerol stock of *E. coli* was inoculated into 2 mL of LB, LBM, SOB, SOC, TB, TH, or TSB. Following 7 h incubation at 37 °C, 1 mL of each culture was taken and centrifuged at 11,000 g for 5 min at RT. The cell pellet was re-suspended in 200 μL of 1× RB. Cleavage reactions were then conducted by mixing 41 μL of 1× RB, 5 μL of each CIM-EC, 1 μL of 2.5 μM FS1, 1 μL of 2.5 μM EC1T and 2 μL of 2× RB. Each reaction mixture was incubated at RT for 60 min, followed by 10% dPAGE analysis as described above.

### 3.9. Comparison of the Cleavage Activity of EC1T in the Presence of Different Divalent Metals

First, stocks of 2× RBʹ (100 mM HEPES, 300 mM NaCl, pH 7.5) and 150 mM MCl_2_ (M = Cd, Co, Mg, Mn, Ni, Cu, Zn and Ca) were prepared. The CIM-EC was also prepared from *E. coli* grown in SOB in the same way as described immediately above except for the use of 1× RBʹ instead of 1× RB. The cleavage reactions as shown in Figure 3A were set up by mixing 15.5 μL of water, 22.5 μL of 2× RBʹ, 5 μL of a relevant MCl_2_ stock, 1 μL of 2.5 μM FS1, 1 μL of 2.5 μM EC1T, and 5 μL of the CIM-EC. The cleavage reactions as shown in Figure 3B were set up similarly except that the volume of water and 150 mM BaCl_2_ were co-varied to achieve a final [BaCl_2_] of 0, 1, 5, 7.5, 10, 15, 20, 25 and 50 mM. Each reaction mixture was incubated at RT for 60 min, followed by 10% dPAGE analysis as described above. 

### 3.10. Comparison of the Cleavage Activity of EC1T at Different Reaction Temperature

A 2× RB_Ba_ stock (100 mM HEPES, 300 mM NaCl, 30 mM BaCl_2_, pH 7.5) was first prepared. Five cleavage reaction mixtures were then set up by mixing 19.5 μL of water, 22.5 μL of 2× RB_Ba_, 1 μL of 2.5 μM FS1, 1 μL of 2.5 μM EC1T, and 5 μL of the CIM-EC prepared with 1× RB_Ba_. These mixtures were incubated, respectively, at 4, 15, 23, 37 and 50 °C for 60 min, followed by 10% dPAGE analysis as described above.

### 3.11. Comparison of the Cleavage Activity of EC1T at Different pH

A series of 2× RB_Ba_ʹ stock (300 mM NaCl, 30 mM BaCl_2_, along with a chosen buffering agent at 100 mM) were first prepared with pH being varied from 5.0 to 9.0 at an increasing interval of 0.5 units. MES was used for pH 5.0, 5.5 and 6.0; HEPES was used for pH 6.5, 7.0, 7.5 and 8.0; Tris was used for pH 8.5 and 9.0. The cleavage reactions were then conducted in a similar fashion as described in the section immediately above. Note that the CIM-EC for a given pH was prepared with a relevant 1× RB_Ba_ʹ.

### 3.12. Comparison of the Cleavage Activity of EC1T at Varying FS1/EC1T Ratios

Stocks of EC1T at 2.5, 5, 12.5, 25, 62.5, 125, and 250 μM were first prepared. Cleavage reactions were then conducted by mixing 19.5 μL of water, 22.5 μL of 2× RB_Ba_, 1 μL of 2.5 μM FS1, 1 μL of a given EC1T stock, and 5 μL of the CIM-EC prepared with 1× RB_Ba_. Each reaction mixture was incubated at RT for 60 min, followed by 10% dPAGE analysis as described above. 

### 3.13. Specificity Test

Five Gram-negative bacteria (*P. peli*, *Y. rukeri*, *H. alvei*, *A. xylosoxidans* and *E. coli*) and four Gram-positive bacteria (*L. mesenteroides*, *L. planturum*, *P. acidilactici* and *B. subtilis*) were tested in this experiment. Each bacterium was cultured in SOB for a different period of time until the OD600 reached ~1. The CIM was then prepared with 1× RB_Ba_ and tested with EC1T/FS1 under the optimal reaction condition (50 mM HEPES, pH 7.5, 150 mM NaCl and 15 mM BaCl_2_, room temperature, EC1T/FS1 = 50/1). Each reaction mixture was incubated at RT for 60 min, followed by 10% dPAGE analysis as described above.

### 3.14. Detection Sensitivity

First, a single colony of *E. coli* from an agar plate was taken, inoculated into 2 mL of SOB and grown for 14 h at 37 °C with shaking at 250 rpm. 10-fold serial dilution was then carried out as follows: 100 μL of the 14-h culture was mixed with 900 μL of fresh SOB. 100 μL of the diluted culture was again taken and mixed with 900 μL of fresh SOB. This process was repeated 7 times. 100 µL of the final dilution were plated onto a TSB agar plate (done in triplicate), which was incubated at 37°C for 15 h. Colonies in each plate were counted; the average number of colonies from the three plates was taken as the number of cells for this final dilution. This number was then used to calculate the number of cells for the other dilutions. 500 μL of each dilution was taken and centrifuged at 11,000 g for 5 min at RT. The cell pellet was re-suspended in 100 μL of 1× RB_Ba_ and used as the CEM-EC for this experiment (done in triplicate). 

Cleavage reactions concerning EC1T/FS1 were set up and monitored as follows: 19.5 μL of water, 22.5 μL of 2× RB_Ba_, 1 μL of 2.5 μM FS1, 1 μL of 125 μM EC1T were mixed in a quartz crystal cuvette, which was placed in a fluorimeter (Cary Eclipse Fluorescence Spectrophotometer; excitation wavelength = 488 nm and emission wavelength = 520 nm) set at RT. Fluorescence intensity was recorded every minute for 5 min; 5 μL of a relevant CIM-EC was then added into the cuvette and the solution was quickly mixed by pipetting the mixture up and down a few times. Following this step, the fluorescence intensity of the solution was recorded for 55 more minutes. All the reactions were conducted in 3 replicates and the average data are shown in Figure 6A. The final reaction mixture was also taken and analyzed by 10% dPAGE and data are shown in Figure 6B.

Cleavage reactions concerning RFD-EC1 were set up and monitored similarly: 20.5 μL of water, 22.5 μL of 2× RB_Ba_, 1 μL of 2.5 μM RFD-EC1 was mixed in a cuvette. After reading fluorescence intensity for 5 min, 5 μL of a relevant CIM-EC was then added, followed by fluorescence intensity reading for 55 more minutes (Figure 6C). The final reaction mixture was also analyzed by 10% dPAGE (Figure 6D).

### 3.15. Single Cell Detection via Culturing

For isolating a single cell we followed our previously reported protocol [12]. Briefly, a glycerol stock containing 2 CFU/mL of *E. coli* was prepared. 100 μL of this stock was distributed to 10 culture tubes each with 2 mL of SOB. Since the concentration of the stock was 2 CFU/mL, only 2 out of the 10 tubes contained a single seeding cell (2 CFU/mL × 0.1 mL = 2). All the tubes were incubated at 37 °C with shaking at 250 rpm. At 2, 4, 6, 8 and 10 h, 200 μL of culture was harvested from each culture tube and CIMs were prepared (40 μL of 1× RB_Ba_ was used to dissolve the cell pellet). All the tubes were further incubated for 20 h to identify the two tubes containing *E. coli* cell (the culture in these tubes turned turbid while that in other 8 tubes stayed clear). Each CIM from *E. coli*-containing tubes was used to initiate the cleavage reaction by mixing 19.5 μL of water, 22.5 μL of 2× RB_Ba_, 1 μL of 2.5 μM FS1, 1 μL of 125 μM EC1T, and 5 μL of a relevant CIM. The reaction and dPAGE analysis procedures were same as described above.

## 4. Conclusions

We recently described an RNA-cleaving fluorogenic DNAzyme, named RFD-EC1, which is active in the presence of the crude extracellular mixture (CEM) of the model Gram-negative bacterium *E. coli* [12,13,14]. RFD-EC1 was found to be highly active with CEM of *E. coli* but inactive with CEMs from a host of other Gram-negative and Gram-positive bacteria, and thus, RFD-EC1 can be used to develop a simple, “mix-and-read” fluorescence assay to achieve selective detection of *E. coli*. However, several parameters that are particularly relevant to the performance of this assay remained to be investigated. In this study we sought to establish a *trans*-acting DNA catalyst that cleaves an external substrate, optimize the reaction conditions that best support the catalytic activity of the DNAzyme, and determine the culturing conditions that enable the quickest detection of a single live bacterial cell. 

The *trans*-acting DNAzyme was successfully established by segregating the substrate sequence domain from the sequence of the original DNA library. Also the two fixed sequence domains flanking the random-sequence domain could be removed without affecting the catalytic performance of the DNAzyme. The shortened, *trans*-acting DNAzyme, named EC1T, now contains 70 nucleotides.

Originally, the DNAzyme was isolated to cleave in the presence of the crude extracellular mixture (CEM) of *E. coli* and it has been determined that the target that activates the DNAzyme is a protein molecule based on the observation that the treatment of the CEM with proteases abolishes the DNAzyme activity [12]. Although the identity of this target is yet to be determined, we found that the target protein is much more abundant intracellularly and could be retrieved with a simple heating step (50 °C; 15 minutes). This led us to the use of the crude intracellular mixture (CIM) as the target of detection, translating into a better assay sensitivity.

Our results revealed that the nutritional factors in culture media played a vital role in growing the cells in faster rate (varying by as much as ~25-fold) with super Optimal Broth (SOB) which can substantially reduce the time required for single cell detection. 

In order to establish an optimal reaction condition for EC1T, we examined the following reaction parameters: choice of divalent metal ions, reaction temperature and pH as well as the ratio between the substrate and the DNAzyme. Although EC1T was found to be active in the absence of any divalent ion, it exhibited much stronger activity in the presence of Ba^2+^, Ca^2+^, Mn^2+^ or Mg^2+^. We chose Ba^2+^ as the divalent metal ion cofactor because this metal ion does not impose any fluorescence quenching effect. The DNAzyme was originally derived at room temperature (~23 °C) and a solution pH of 7.5 and therefore it was not surprising that EC1T exhibited the strongest activity at 23 °C and pH 7.5. We further found that when the concentration of FS1 was kept at 50 nM, 2.5 μM EC1T was required to reach the optimal cleavage activity. All the above optimization experiments led to the establishment of the optimal reaction condition for EC1T: 50 mM HEPES, 150 mM NaCl, 15 mM BaCl_2_, pH 7.5, DNAzyme: substrate ratio = 50:1.

Under the above optimal reaction condition, the *trans*-acting system was able to detect 10^5^ cells when the reaction was monitored in a fluorimeter. If the reaction mixture was analyzed by dPAGE (which separates the reaction product from the substrate), the system can detect 10^4^ cells. When the original RFD-EC1 was used for the assay, the detection sensitivity was further improved: the fluorimeter method was able to detect 10^4^ cells while the dPAGE method was able to detect as low as 10^3^ cells. Importantly, the optimized assay did not compromise the specificity. 

With a culturing step, the optimized assay is able to achieve the detection of *E. coli* from a single colony forming unit in 4–6 hours (dependent on the method of choice), which represents a significant deduction in time (12 h) required by the same probe under unoptimized conditions. Overall, we have significantly improved the performance of our DNAzyme probe and demonstrate the utility of such probes as simple biosensors to achieve sensitive and speedy detection of bacterial pathogens. 
